# Characterization of the FKBP12-Encoding Genes in *Aspergillus fumigatus*

**DOI:** 10.1371/journal.pone.0137869

**Published:** 2015-09-14

**Authors:** Katie Falloon, Praveen R. Juvvadi, Amber D. Richards, José M. Vargas-Muñiz, Hilary Renshaw, William J. Steinbach

**Affiliations:** 1 Duke University School of Medicine, Durham, NC, United States of America; 2 Department of Pediatrics, Division of Pediatric Infectious Diseases, Duke University Medical Center, Durham, NC, United States of America; 3 Department of Molecular Genetics and Microbiology, Duke University Medical Center, Durham, NC, United States of America; Universidade de Sao Paulo, BRAZIL

## Abstract

Invasive aspergillosis, largely caused by *Aspergillus fumigatus*, is responsible for a growing number of deaths among immunosuppressed patients. Immunosuppressants such as FK506 (tacrolimus) that target calcineurin have shown promise for antifungal drug development. FK506-binding proteins (FKBPs) form a complex with calcineurin in the presence of FK506 (FKBP12-FK506) and inhibit calcineurin activity. Research on FKBPs in fungi is limited, and none of the FKBPs have been previously characterized in *A*. *fumigatus*. We identified four orthologous genes of FKBP12, the human FK506 binding partner, in *A*. *fumigatus* and designated them *fkbp12-1*, *fkbp12-2*, *fkbp12-3*, and *fkbp12-4*. Deletional analysis of the four genes revealed that the Δ*fkbp12-1* strain was resistant to FK506, indicating FKBP12-1 as the key mediator of FK506-binding to calcineurin. The endogenously expressed FKBP12-1-EGFP fusion protein localized to the cytoplasm and nuclei under normal growth conditions but also to the hyphal septa following FK506 treatment, revealing its interaction with calcineurin. The FKBP12-1-EGFP fusion protein didn’t localize at the septa in the presence of FK506 in the *cnaA* deletion background, confirming its interaction with calcineurin. Testing of all deletion strains in the *Galleria mellonella* model of aspergillosis suggested that these proteins don’t play an important role in virulence. While the Δ*fkbp12-2* and Δ*fkbp12-3* strains didn’t show any discernable phenotype, the Δ*fkbp12-4* strain displayed slight growth defect under normal growth conditions and inhibition of the caspofungin-mediated “paradoxical growth effect” at higher concentrations of the antifungal caspofungin. Together, these results indicate that while only FKBP12-1 is the bona fide binding partner of FK506, leading to the inhibition of calcineurin in *A*. *fumigatus*, FKBP12-4 may play a role in basal growth and the caspofungin-mediated paradoxical growth response. Exploitation of differences between *A*. *fumigatus* FKBP12-1 and human FKBP12 will be critical for the generation of fungal-specific FK506 analogs to inhibit fungal calcineurin and treat invasive fungal disease.

## Introduction

Medical advancement, especially in the fields of transplantation and oncology, has led to a growing population of immunosuppressed patients. Unfortunately, as this population has expanded, the incidence of infections in these patients has also increased and invasive fungal infections are a leading cause of infection-related mortality in the immunosuppressed [1–5]. Chief among these infections is invasive aspergillosis, largely caused by *Aspergillus fumigatus*, which kills 40–60% of those it infects [2, 3, 6–8]. Given the relative ineffectiveness of current antifungal treatment options, an improved understanding of invasive aspergillosis, coupled with novel therapeutic agents, is needed [9].

Paradoxically, the immunosuppressants that accommodate organ transplantation, yet render patients susceptible to opportunistic infections, also possess the ability to halt invasive aspergillosis [9–11]. Epidemiologically, patients on agents that inhibit the Ca^2+^/calmodulin (CaM) dependent protein phosphatase calcineurin, such as cyclosporine A and FK506 (tacrolimus), have been shown to be less likely to suffer invasive fungal infections than those receiving other forms of immunosuppression [12, 13]. Additionally, *in vitro* testing shows cyclosporine A and FK506 both interfere with fungal growth and virulence [14].

Calcineurin inhibitors function by first forming complexes with immunophilins, highly conserved peptidyl-prolyl cis-trans isomerases that serve as chaperones in protein folding in organisms from fungi to humans [15–20]. The immunophilins can be further classified into cyclophilins, which bind to cyclosporine A, and FK506-binding proteins (FKBPs), which bind to FK506 or rapamycin [21–25]. Immunosupressant-immunophilin complexes then bind to calcineurin between its catalytic (CnaA) and regulatory (CnaB) subunits to exert their inhibitory effects [15, 16, 26, 27]. In humans, this binding prevents activation of the immune system [21, 28, 29]. In *A*. *fumigatus*, binding prevents a number of functions important for fungal pathogenesis, including regulation of stress response, cation homeostasis, cell wall integrity, and virulence [30–35]. Given this unique mechanism of antifungal activity, as well as the synergism of calcineurin inhibitors with standard antifungals and antifungal activity against drug resistant strains, the calcineurin pathway is an optimal target for drug development [9, 11, 14, 36, 37]. With appropriate chemical modifications, it is possible that a calcineurin inhibitor could be designed for fungal-specific targeting, leaving human calcineurin, and by extension the human immune system, untouched [9]. Therefore, it is important to gain a better understanding of one of the key binding partners of calcineurin, FKBP.

Work on FKBPs in mammals has been extensive, and mammalian FKBPs have been shown to interact with TGF-β as well as with calcium release channels (ryanodine receptors and inositol 1,4,5 triphosphate receptors) via calcineurin and mTOR [38–44]. On the contrary, exploration of FKBPs in fungi has been limited. Work in the model organisms *Saccharomyces cerevisiae* and *Neurospora crassa* shows no essential role for the FKBPs [45, 46], and orthologs of FKBP12 in both fungi mediated resistance to FK506 and rapamycin [45–48]. Studies in the plant pathogens *Botrytis cinerea* and *Fusarium fujikuroi* also demonstrated a role for fungal orthologs of FKBPs in FK506 and rapamycin resistance [49–51]. In human pathogenic fungi, deletions of the FKBP12 ortholog *frr1* in *Cryptococcus neoformans* and disruptions in the FKBP12 ortholog *fkbA* in *Mucor circinelloides* have also led to FK506 and rapamycin resistance [52–55]. However, no studies have focused on FKBPs in one of the most common invasive fungal pathogens, *A*. *fumigatus*.

In the present study, we identified four orthologs of human FKBP12 in *A*. *fumigatus* and characterized their roles in hyphal growth, FK506 sensitivity and virulence. Of the four FKBP12s, FKBP12-1 is critical to target in future drug development, and exploitation of the difference between it and human FKBP12 could prove important in the generation of fungal-specific FK506 analogs.

## Materials and Methods

### Strains, media, and growth conditions

The *A*. *fumigatus akuB* ^*KU80*^ *pyrG* ^*-*^ uracil/uridine auxotroph strain was used as the recipient strain in the construction of the Δ*fkbp12-1*, Δ*fkbp12-2*, Δ*fkbp12-3*, and Δ*fkbp12-4* deletion strains [56, 57]. It was also used in the construction of the *fkbp12-1-egfp* expression strain in the *pyrG* ^*-*^ background. The *fkbp12-1-egfp pyrG* ^*-*^ strain was then used as the recipient strain in the generation of the *fkbp12-1-egfp*Δ*cnaA* strain. The *A*. *fumigatus akuB* ^*KU80*^ strain was used as the recipient strain in construction of the *fkbp12-1-egfp* strain, as well as the wild-type control for all experiments [57]. The *A*. *fumigatus* Δ*fkbp12-1* strain was used as the recipient strain in the generation of the Δ*fkbp12-1*Δ*fkbp12-2* double deletion strain. All *A*. *fumigatus* cultures were grown on glucose minimal media (GMM) at 37°C as previously described, unless otherwise specified [58]. *Escherichia coli* DH5α competent cells (New England Biolabs, Ipswich, MA) were used for cloning and grown on LB media supplemented with appropriate antibiotics at 37°C.

### Construction of FKBP12 single and double deletion strains

With a focus on the role of *A*. *fumigatus* FKBPs in mediating antifungal resistance or pathogenesis, we constructed deletion strains of all FKBP12-encoding genes (Δ*fkbp12-1*, Δ*fkbp12-2*, Δ*fkbp12-3*, and Δ*fkbp12-4*). Primers used for the construction of the various deletion cassettes are listed in the S1 Table. The Δ*fkbp12-1* strain was constructed via replacement of the 637 bp *fkbp12-1* gene (*fkbp1*/Afu6g12170, www.aspergillusgenome.org) with the 3.0 kb *A*. *parasiticus pyrG* gene to serve as a selectable marker to complement the uracil/uridine auxotrophy of *akuB* ^*KU80*^ [31]. Approximately 1 kb of flanking upstream sequence of *fkbp12-1* was PCR amplified from *A*. *fumigatus* strain AF293 genomic DNA and cloned into the pCDF-Duet-1 vector (Novagen EMD Millipore, Billerica, MA), using the BamHI and EcoRI sites. Fusion PCR was used to generate the ~4.0 kb sequence containing the *A*. *parasiticus pyrG* gene and ~1 kb of flanking downstream sequence of *fkbp12-1*, which was also cloned in the pCDF-Duet-1 vector using the EcoRI and SacI sites. The resulting replacement construct plasmid was used as a template to create the ~4.7 kb PCR amplicon for use in transformation into the *akuB* ^KU80^ *pyrG* ^*-*^ strain.

The Δ*fkbp12-2* strain was constructed via replacement of the 709 bp *fkbp12-2* gene (*fkbp2*/Afu4g04020, www.aspergillusgenome.org) with the 3.0 kb *A*. *parasiticus pyrG* gene. Approximately 1 kb of flanking upstream and downstream sequences were PCR amplified from AF293 genomic DNA and cloned into the pJW24 plasmid, using the SalI and EcoRI sites for the upstream sequence and the BamHI and NotI sites for the downstream sequence. The resulting replacement construct plasmid was then linearized via NotI digestion to yield the final construct for transformation into the *akuB* ^KU80^ *pyrG* ^*-*^ strain.

The Δ*fkbp12-3* strain was constructed via replacement of the 485 bp *fkbp12-3* gene (*fkbp3*/Afu2g03870, www.aspergillusgenome.org) with the 3.0 kb *A*. *parasiticus pyrG* gene. Approximately 1 kb of flanking upstream and 608 bp of flanking downstream sequences were PCR amplified from AF293 genomic DNA and cloned into the pJW24 plasmid, using the NotI and XbaI sites for the upstream sequence and the EcoRI and SalI sites for the downstream sequence. The resulting replacement construct plasmid was used as a template to create the ~4.7 kb PCR amplicon for use in transformation into the *akuB* ^KU80^ *pyrG* ^*-*^ strain.

The Δ*fkbp12-4* strain was constructed via replacement of the 1653 bp *fkbp12-4* gene (*fkbp4*/Afu6g08580, www.aspergillusgenome.org) with the 3.0 kb *A*. *parasiticus pyrG* gene. Approximately 1 kb of flanking upstream and downstream sequences were PCR amplified from AF293 genomic DNA and cloned into the pJW24 plasmid, using the SalI and EcoRI sites for the upstream sequence and the NotI and SacI sites for the downstream sequence. The resulting replacement construct plasmid was then linearized via digestion with SalI and SacI to yield the construct for use in transformation into the *akuB* ^KU80^ *pyrG* ^*-*^ strain.

For strains Δ*fkbp12-1* through Δ*fkbp12-4*, transformants were selected for growth in the absence of uracil/uridine supplementation.

The Δ*fkbp12-1*Δ*fkbp12-2* double deletion strain was constructed via replacement of the 709 base pair *fkbp12-2* gene (*fkbp2*/Afu4g04020, www.aspergillusgenome.org) with the 4.4 kb hygromycin B resistance (*hph)* cassette. Approximately 1 kb of flanking upstream and downstream sequences were PCR-amplified from AF293 genomic DNA and cloned into the pUCGH plasmid, using the HindIII and SbfI sites for the upstream sequence and the EcoRV and NotI sites for the downstream sequence. The resulting replacement construct plasmid was then linearized via digestion with NotI, yielding the construct for use in transformation into the Δ*fkbp12-1* strain. Transformants were selected for resistance to hygromycin B. Primers utilized to construct this strain are listed in the S1 Table.

To construct the *fkbp12-1-egfp* strain, 384 bp of the 637 bp *fbkp12-1* gene (*fkbp1*/Afu6g12170, www.aspergillusgenome.org) and ~1 kb of the *fkbp12-1* terminator sequence were PCR amplified from AF293 genomic DNA and cloned into the pUCGH plasmid at the N-terminus of *egfp*, using the KpnI and BamHI sites for the gene and the SbfI and HindIII sites for the terminator sequence. The plasmid was then sequenced to confirm accuracy of the partial sequence of the *fkbp12-1* cloned and finally linearized via single restriction enzyme digestion with KpnI. The construct was transformed into the *A*. *fumigatus akuB* ^*KU80*^ strain. Transformants were selected for resistance to hygromycin B. All primers utilized to construct the GFP strain are listed in the S4 Table.

To construct the *fkbp12-1-egfp*Δ*cnaA* strain, first 384 bp of the 637 bp *fbkp12-1* gene (*fkbp1*/Afu6g12170, www.aspergillusgenome.org) and ~1 kb of the *fkbp12-1* terminator sequence were PCR amplified from AF293 genomic DNA and cloned into the pUCGH plasmid at the N-terminus of *egfp*, using the KpnI and BamHI sites for the gene and the SbfI and HindIII sites for the terminator sequence. The plasmid was then sequenced to confirm accuracy of the partial sequence of the *fkbp12-1* cloned and finally linearized via single restriction enzyme digestion with KpnI. The construct was transformed into the *A*. *fumigatus akuB* ^KU80^ *pyrG* ^*-*^ strain. Next, the 3.0 kb *A*. *parasiticus pyrG* gene was used to replace the 1.9 kb *cnaA* gene (*calA*/Afu5g09360, www.aspergillusgenome.org) as previously described [31] and the resulting replacement construct was transformed into the *akuB* ^KU80^ *pyrG* ^*-*^ *fkbp12-1-egfp* strain.

For all 6 strains, generation of the fungal protoplasts and polyethylene glycol-mediated transformation was performed as previously described [31]. Transformants were initially screened by PCR with primers designed to amplify the deleted genes and also with primers flanking the deleted gene to verify homologous recombination. All primers used to verify proper integration in the deletion strains are listed in the S2 Table. Confirmation of gene deletion was performed via Southern analysis using a digoxigenin labeling system (Roche Molecular Biochemicals, Mannheim, Germany) for all deletion strains. The primers used to generate the probes used for Southern analysis in each strain are listed in the S3 Table. All primers used to verify proper integration in the *egfp* strains are listed in the S5 Table. Fluorescence microscopy served as the second confirmatory test for the FKBP12-1-EGFP and FKBP12-1-EGFPΔ*cnaA* strains.

### Radial Growth

Radial growth on solid media was quantified as previously described for all deletion strains and the FKBP12-1-EGFP strain [31]. All assays were performed in triplicate. To further validate the slight growth defect observed with the Δ*fkbp12-4* strain, the assays were performed in triplicate in two independent experiments. Statistical comparison was performed using Graph Pad Prism (San Diego, CA).

### Antifungal and immunosuppressant susceptibility testing

FK506, cyclosporine A, and caspofungin were obtained as commercial products. Rapamycin was obtained from the National Cancer Institute. An inoculum of 10 μL of 1x10^6^ conidia/mL (10^4^ conidia) was spotted onto GMM plates supplemented with either FK506 (100 ng/mL) or cyclosporine A (10 μg/mL). Testing for caspofungin sensitivity was performed with GMM plates supplemented with either 1 μg/mL or 4 μg/mL of caspofungin and growth was observed after 5 days [34]. Susceptibility to caspofungin was also analyzed in 96 well plates using RPMI 1640 liquid media (RPMI; Roswell Park Memorial Institute) supplemented with either 1 μg/mL or 4 μg/mL of caspofungin. Hyphal growth was visualized microscopically after incubation at 37°C for 24 and 48 hours. Spotting on GMM supplemented with both FK506 (100 ng/mL) and caspofungin (1 μg/mL) was performed to assess the combined effect on antifungal resistance. Susceptibility to FK506 was also analyzed in 96 well plates using RPMI 1640 media and 100 ng/mL of FK506 [59]. Hyphal growth was visualized microscopically after incubation at 37°C for 24 and 48 hours. Given the high MIC of the drug, resistance to rapamycin was analyzed in 96 well plates using RPMI media and 100 μg/mL of rapamycin [14]. Hyphal growth was visualized microscopically after incubation at 37°C for 24 hours. All drug testing was performed in triplicate.

### Virulence Testing

Twenty larvae of the waxmoth *Galleria mellonella* were injected with 5 μl of 1 x 10^8^ spores/ml (total inoculum of 2 x 10^5^ spores) of the wild-type or the respective FKBP12 deletion strains. Infected larvae were incubated at 37°C with survival scored daily for 5 days [60]. Data from this trial was plotted on a Kaplan-Meier curve with log rank pair-wise comparison and statistical analysis was performed using Graph Pad Prism (San Diego, CA).

### Light and Fluorescence Microscopy

Conidia of the *fkbp12-1-egfp* strain were cultured for 18–20 hours at 37°C in petri dishes containing coverslips immersed in 10 mL of GMM liquid media [32]. To assess localization following exposure to FK506, conidia of the *fkbp12-1-egfp* strain were cultured for 18–20 hours at 37°C in 60x15 mm petri dishes containing coverslips immersed in 10 mL GMM liquid media supplemented with 100 ng/mL of FK506. Following incubation, spores were adherent to the coverslip and could be visualized via microscopy. To confirm that the septal localization observed was due to interaction with calcineurin, both experiments were repeated with the *fkbp12-1-egfp*Δ*cnaA* strain. Fluorescence microscopy was performed using an Axioskop 2 plus microscope (Zeiss) equipped with AxioVision 4.6 imaging software with Nomarski optics (Differential Interference Contrast) and fluorescence. For nuclear staining, the *fkbp12-1-egfp* and wild type strains were cultured in GMM liquid medium on coverslips for 18–20 hours and stained with propidium iodide. Briefly, the cultures were washed in 50 mM PIPES (pH 7.0) for 5 minutes, fixed in 8% formaldehyde with 0.2% Triton X-100 for 45 minutes at 25°C, washed in 50 mM PIPES (pH 7.0) for 10 minutes and treated with RNase (100 μg/ml) for 60 minutes at 37°C. After washing with 50 mM PIPES (pH 7.0) for 20 minutes, the fixed sample was stained with propidium iodide solution (12.5 μg/ml) in 50 mM PIPES (pH 7.0) for 5 minutes, washed again with 50 mM PIPES (pH 7.0) for 20 minutes and observed under the fluorescence microscope.

## Results

### Identification and phylogenic analysis of putative *A*. *fumigatus* FKBP12 orthologs

**Fig 1:**
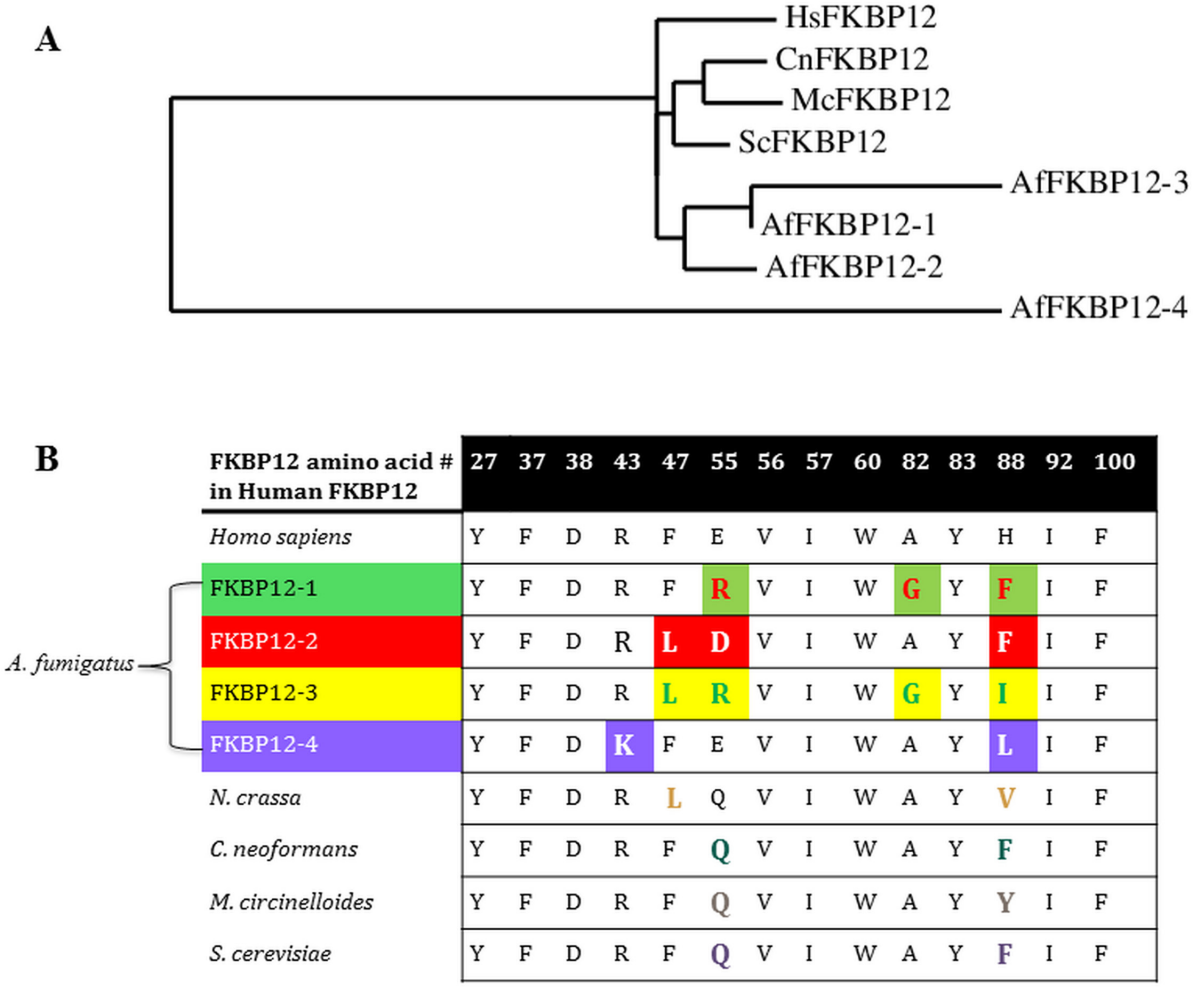
Phylogenetic analysis of FKBP12 proteins and multiple sequence alignment comparing residues important for FK506-FKBP12 binding. **(A)** Phylogram showing orthologous FKBP12 proteins from Human (HsFKBP12), *S*. *cerevisiae* (ScFKBP12), *C*. *neoformans* (CnFKBP12), *M*. *circinelloides* (McFKBP12) and *A*. *fumigatus* (AfFKBP12-1, AfFKBP12-2, AfFKBP12-3 and AfFKBP12-4). Phylogenic analysis was performed using the respective amino acid sequences aligned with MUSCLE (v3.8.31) implemented in the PhyML program (v3.1/3.0 aLRT). Graphical representation of the phylogenetic tree was performed with TreeDyn (v198.3). *A*. *fumigatus* FKBP12 proteins are designated as AfFKBP12-1, AfFKBP12-2, AfFKBP12-3 and AfFKBP12-4, respectively. **(B)** 14 residues are known to be important for binding FKBP12 to FK506. Residues distinct from human FKBP12 are highlighted in a different color for each *A*. *fumigatus* FKBP12 and for each species-specific FKBP12 homolog.

In order to characterize the role of FKBP12 orthologs in *A*. *fumigatus*, BLAST analysis was used to identify the four putative orthologs of human *fkbp12* (www.aspergillusgenome.org). Four FKBPs (*fkbp12-1*, *fkbp12-2*, *fkbp12-3* and *fkbp12-4)* were identified. Comparison with human FKBP12 revealed that FKBP12-1 had the greatest sequence similarity (55% identity, 74% similarity, http://www.ebi.ac.uk/Tools/psa/emboss_needle), followed by FKBP12-2 (47% identity, 68% similarity, http://www.ebi.ac.uk/Tools/psa/emboss_needle), FKBP12-3 (35% identity, 50% similarity, http://www.ebi.ac.uk/Tools/psa/emboss_needle) and finally FKBP12-4 (10% identity, 14% similarity, http://www.ebi.ac.uk/Tools/psa/emboss_needle). Multiple sequence alignment of the proteins (S1 Fig) and phylogenetic analysis (Fig 1A) showed that the FKBP12 proteins from human and other fungal species were closely related. While FKBP12-1, FKBP12-2 and FKBP12-3 were grouped together, FKBP12-4 may belong to a separate clade and diverged from the other members. Based on the sequence similarity between FKBP12-1 and FKBP12-2, it is possible that FKBP12-2 may have been formed due to gene duplication. Comparison of amino acids at the 14 residues previously identified as key for binding to FK506 [61] revealed that FKBP12-1 differed from human FKBP12 at three of the 14 sites, FKBP12-2 at three of the 14 sites, FKBP12-3 at four of the 14 sites, and FKBP12-4 at two of the 14 sites (Fig 1B). The three substitutions in *A*. *fumigatus* FKBP12-1 involved the replacement of an acidic amino acid with a basic one (arginine for glutamate, position 55), the replacement of one small nonpolar amino acid with another (glycine for alanine, position 82), and the replacement of a basic amino acid with a nonpolar and bulky one (phenylalanine for histidine, position 88). As is illustrated in Fig 1B, FKBP12 orthologs from other species are mutated only at two residues.

The modifications in FKBP12-2, which shares the next most sequence similarity to human FKBP12, include replacement of the neutral phenylalanine with the nonpolar leucine (position 47), replacement of the acidic glutamate with the acidic aspartate (position 55) and replacement of histidine with phenylalanine (position 88). FKBP12-3 differs by four amino acids at similar locations, including replacement of phenylalanine with leucine (position 47), glutamate with arginine (position 55), alanine with glycine (position 82) and histidine with the nonpolar isoleucine (position 88). FKBP12-4, which shares the least sequence similarity to human FKBP12, differs from human FKBP12 at only two of the 14 residues (replacement of arginine with lysine at position 43 and replacement of histidine with leucine at position 88).

### Deletion analysis of the FKBP12 genes in *A*. *fumigatus*

**Fig 2:**
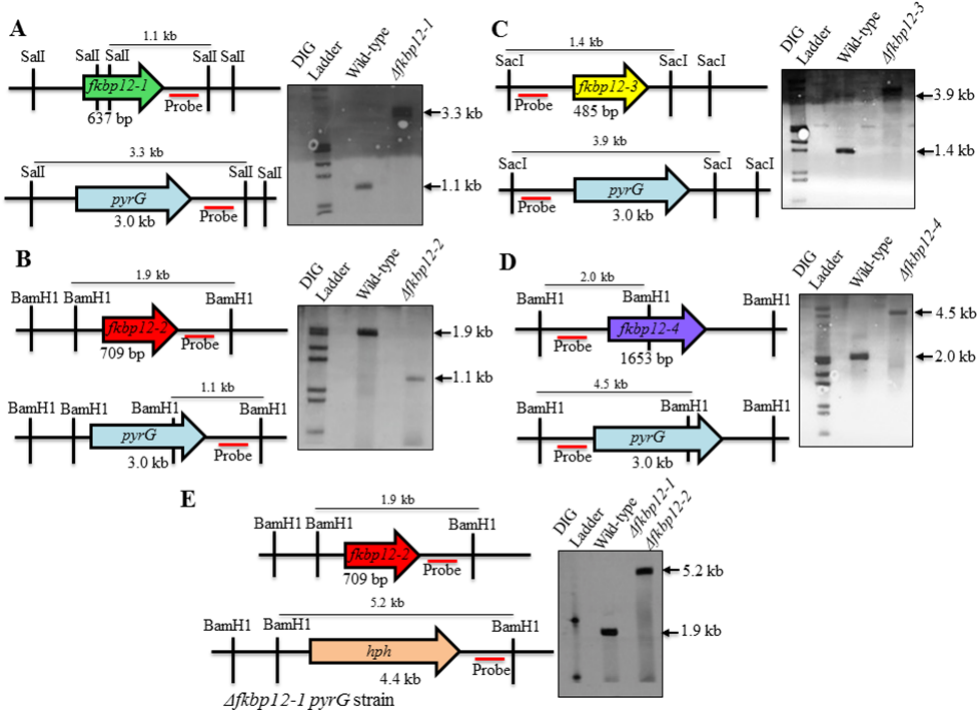
Construction of the FKBP12 deletion strains. **(A) Construction of Δ*fkbp12-1* strain**. In the Δ*fkbp12-1* strain, wild-type *A*. *fumigatus fkbp12-1* (637 bp) was replaced by the 3.0 kb *A*. *parasiticus pyrG* gene. Three of the five strains validated by PCR were then selected for Southern analyses. SalI-digested genomic DNA was probed with the 646 bp probe of the downstream flanking sequence to confirm homologous recombination. Two of the three tested strains demonstrated the expected ~3.3 kb length, which is contrasted with the WT length of ~1.1 kb. Gel used for Southern analysis was 1% agarose. **(B) Construction of Δ*fkbp12-2* strain.** In the Δ*fkbp12-2* strain, wild-type *A*. *fumigatus fkbp12-2* (709 bp) was replaced with the 3.0 kb *A*. *parasiticus pyrG* gene. The strain validated by PCR was then used for Southern analyses. BamHI-digested genomic DNA was probed with the 733 bp probe of the downstream flanking sequence to confirm homologous recombination. The strain demonstrated the expected ~1.1 kb length in contrast with the ~2 kb length in the wild-type strain. Gel used for Southern analysis was 1.5% agarose. **(C) Construction of Δ*fkbp12-3* strain.** In the Δ*fkbp12-3* strain, wild-type *A*. *fumigatus fkbp12-3* (485 bp) was replaced by the 3.0 kb *A*. *parasiticus pyrG* gene. Four of the strains validated by PCR were then selected for Southern analyses. SacI-digested genomic DNA was probed with the 446 bp probe of the downstream flanking sequence to confirm homologous recombination. All four tested strains demonstrated the expected ~3.9 kb length as opposed to the wild-type length of ~1.4 kb. Gel used for Southern analysis was 1% agarose. **(D) Construction of Δ*fkbp12-4* strain.** In the Δ*fkbp12-4* mutant, wild-type *A*. *fumigatus fkbp12-4* (1653 bp) was replaced by the 3.0 kb *A*. *parasiticus pyrG* gene. Four of the strains validated by PCR were then selected for Southern analyses. BamHI-digested genomic DNA was probed with the 677 bp probe of the downstream flanking sequence to confirm homologous recombination. All four tested strains demonstrated the expected ~4.5 kb length as opposed to the wild-type length of ~2.0 kb. Gel used for Southern analysis was 1% agarose. **(E) Construction of Δ*fkbp12-1*Δ*fkbp12-2* strain**. In the Δ*fkbp12-1*Δ*fkbp12-2* strain, wild-type *A*. *fumigatus fkbp12-2* (709 bp) is replaced by the 4.4 kb hygromycin B resistance cassette in the Δ*fkbp12-1* strain. Four of the strains validated by PCR were then selected for Southern analyses. BamHI-digested genomic DNA was probed with the 550 bp probe of the downstream flanking sequence to confirm homologous recombination. All four tested strains demonstrated the expected ~5.2 kb as opposed to the wild-type length of ~1.9 kb.

**Fig 3:**
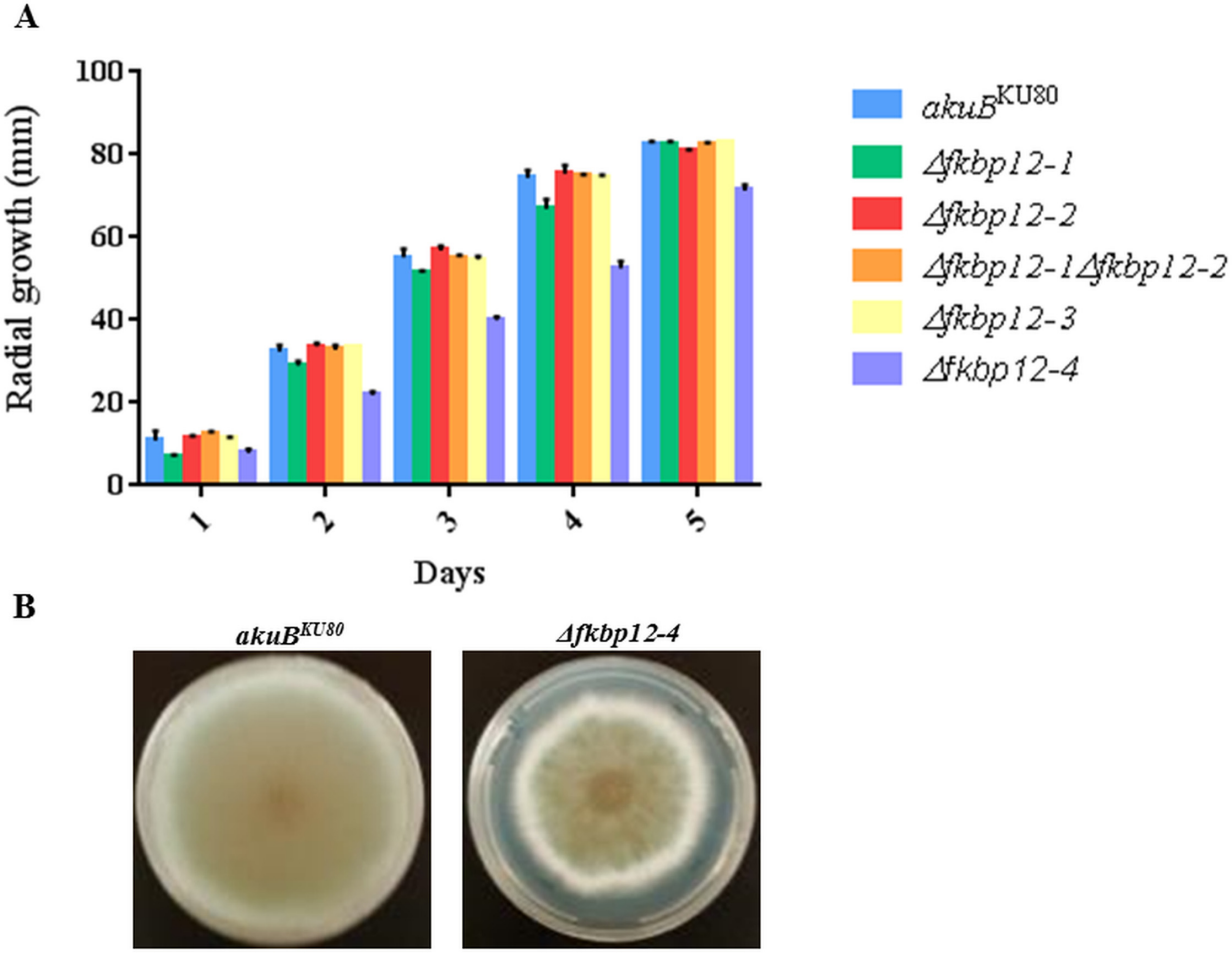
FKBP12-4 is required for complete hyphal growth. **(A)** Growth of *A*. *fumigatus* (10^4^ conidia) on GMM at 37°C for 5 days, with colony diameter measured every 24 hours, revealed no statistically significant difference in growth among Δ*fkbp12-1*, Δ*fkbp12-2*, Δ*fkbp12-1*Δ*fkbp12-2*, Δ*fkbp12-3* and wild-type strains. Δ*fkbp12-4* demonstrated reduced growth rate across all 5 days (p = 0.0161). **(B)** After the 5 day growth period, Δ*fkbp12-4* demonstrated reduced growth compared to wild-type strain but no other obvious phenotypic abnormalities were noted.

Based on the *in silico* analysis, single deletion strains for all four *A*. *fumigatus* FKBP12 orthologs were generated (Fig 2A–2D). In addition, due to the higher homology observed between the FKBP12-1 and FKBP12-2 proteins, a double deletion strain of FKBP12-1 and FKBP12-2 (Δ*fkbp12-1*Δ*fkbp12-2*) was generated to verify any coordinated function between the two proteins (Fig 2E). Successful deletion for each strain was confirmed by PCR (data not shown) and Southern analysis (Fig 2). Various recombinant strains generated in this study are listed in Table 1. Radial growth assays of all the FKBP12 deletion strains revealed them to be non-essential in *A*. *fumigatus* (Fig 3A). Among the respective deletion strains only the Δ*fkbp12-4* strain demonstrated slightly impaired growth under basal conditions compared to the wild-type strain (p = 0.016) (Fig 3A and 3B). Apart from the reduced growth rate, however, there were no other visible growth abnormalities in the strain (Fig 3B). The Δ*fkbp12-1* strain had a statistically significant difference in growth compared to the wild-type strain (p = 0.0405), but by day 5 had reached a similar full growth. All other deletion strains demonstrated radial growth patterns consistent with that seen with the wild-type strain (Fig 3A). In Δ*fkbp12-1*Δ*fkbp12-2*, Δ*fkbp12-2*, and Δ*fkbp12-*3, statistically significant differences in growth were not observed (p = 0.4318, p = 0.2601, p = 0.3138). Thus, of the four FKBP12s, only FKBP12-4 is required for proper growth under basal conditions.

**Table 1:** Strains used in the Present Study.

Strain	Parent Strain	Genotype	Origin
*akuB* ^*KU80*^	CEA17	Wild-type	CBS144-89 (d’Enfert 1996)
*akuB* ^*KU80*^ *pyrG* ^*-*^	CEA17 *pyrG* ^*+*^	*pyrG*	da Silva Ferreira *et al* 2006
Δ*fkbp12-1*	*akuB* ^*KU80*^ *pyrG* ^*-*^	Δ*fkbp12-1*:: *pyrG*	This study
Δ*fkbp12-2*	*akuB* ^*KU80*^ *pyrG* ^*-*^	Δ*fkbp12-2*:: *pyrG*	This study
Δ*fkbp12-3*	*akuB* ^*KU80*^ *pyrG* ^*-*^	Δ*fkbp12-3*:: *pyrG*	This study
Δ*fkbp12-4*	*akuB* ^*KU80*^ *pyrG* ^*-*^	Δ*fkbp12-4*:: *pyrG*	This study
Δ*fkbp12-1*Δ*fkbp12-2*	Δ*fkbp12-1*	Δ*fkbp12-1*:: *pyrG* Δ*fkbp12-2*::*hph*	This study
*fkbp12-1-egfp*	*akuB* ^*KU80*^	*fkbp12-1-egfp*::*hph*	This study
*fkbp12-1-egfp*	*akuB* ^*KU80*^ *pyrG* ^*-*^	*fkbp12-1-egfp*::*hph*	This study
*fkbp12-1-egfpΔcnaA*	*akuB* ^*KU80*^ *pyrG* ^*-*^	*fkbp12-1-egfp*::*hph* Δ*cnaA*::*pyrG*	This study

### FKBP12-1 is the key protein that binds to FK506 and inhibits calcineurin

**Fig 4:**
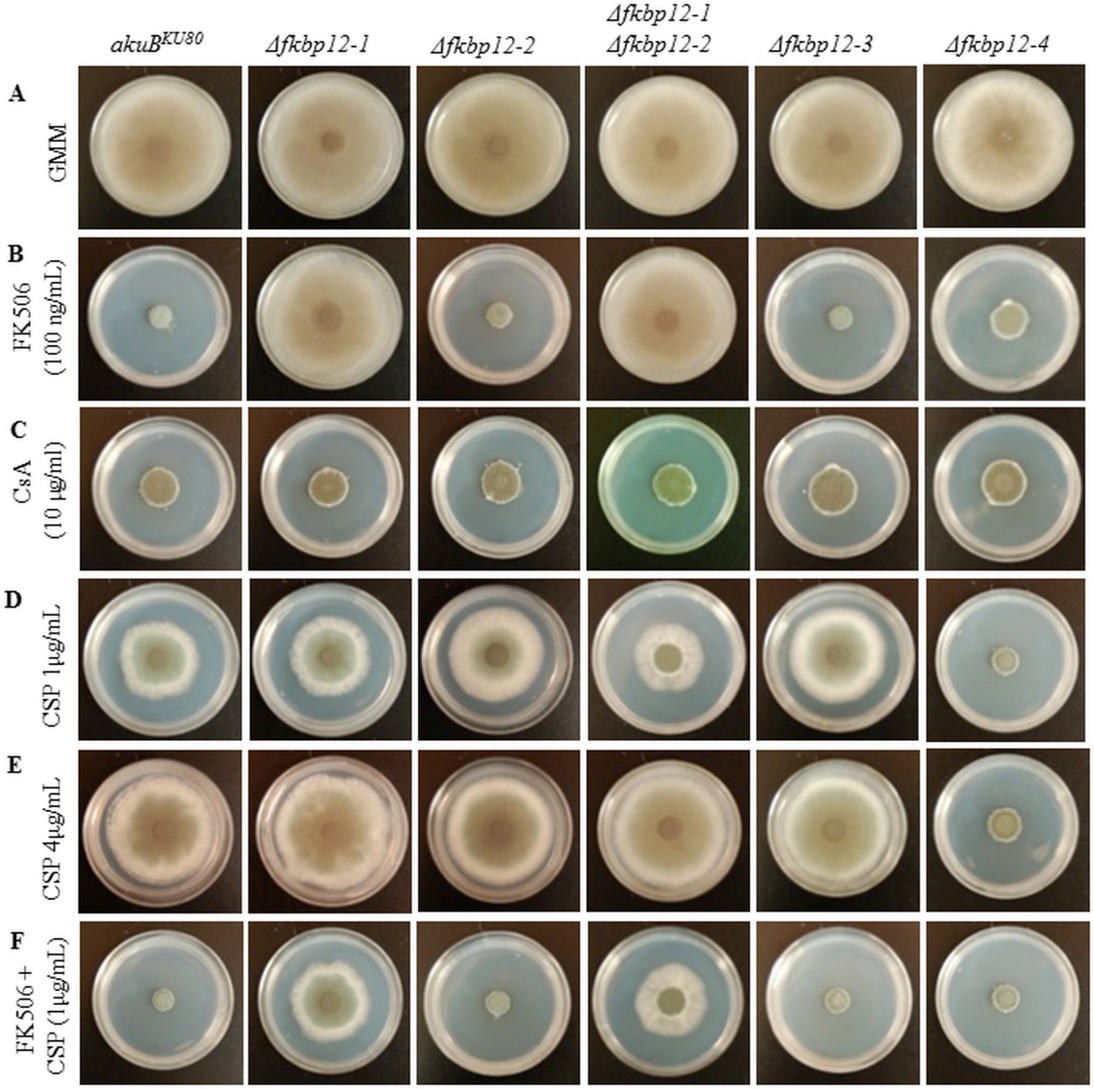
Δ*fkbp12-1* is resistant to FK506 but its response to other antifungal agents is unchanged. **(A)** *A*. *fumigatus* conidia (10^4^ conidia) incubated on GMM at 37°C for 5 days. **(B)** *A*. *fumigatus* conidia (10^4^ conidia) incubated on GMM + 100 ng/mL FK506 at 37°C for 5 days. **(C)** *A*. *fumigatus* conidia (10^4^ conidia) incubated on GMM + 10 μg/mL cyclosporine A (CsA) at 37°C for 5 days. **(D)** *A*. *fumigatus* conidia (10^4^ conidia) incubated on GMM + 1 μg/mL caspofungin (CSP) at 37°C for 5 days **(E)** *A*. *fumigatus* conidia (10^4^ conidia) incubated on GMM + 1 μg/mL caspofungin at 37°C for 5 days **(F)** *A*. *fumigatus* conidia (10^4^ conidia) incubated on GMM + 100 ng/mL FK506 + 1 μg/mL caspofungin at 37°C for 5 days.

Next, in order to determine which of these putative FKBP12 proteins is involved in the binding of FK506 and inhibition of calcineurin function in *A*. *fumigatus*, the respective deletion strains were cultured in the absence or presence of FK506 (100 ng/mL) (Fig 4A and 4B). As shown in Fig 4B, with the exception of the Δ*fkbp12-1* strain and the Δ*fkbp12-1*Δ*fkbp12-2* double deletion strain, all deletion strains showed sensitivity to FK506. Δ*fkbp12-*2 and Δ*fkbp12-*3 showed susceptibility to FK506 comparable to that of the wild-type strain (Fig 4B). These results confirmed that the FK506 resistance observed in the Δ*fkbp12-1*Δ*fkbp12-2* double deletion strain was due to the deletion of *fkbp12-1*. Δ*fkbp12-*4 showed minimal tolerance to FK506, with slightly less sensitivity to the drug than was seen in the wild type strain (Fig 4B). Testing with another immunosuppressant, cyclosporine A, demonstrated susceptibility indistinguishable from the wild-type strain (Fig 4C), indicating that FKBP12-1 specifically binds to FK506 and inhibits calcineurin function. This is expected given the different mechanism of action of cyclosporine A, which binds to cyclophilin A and causes the inhibition of calcineurin. The Δ*fkbp12-*1 strain also demonstrated resistance to rapamycin (100 μg/mL) (data not shown).

**Fig 5:**
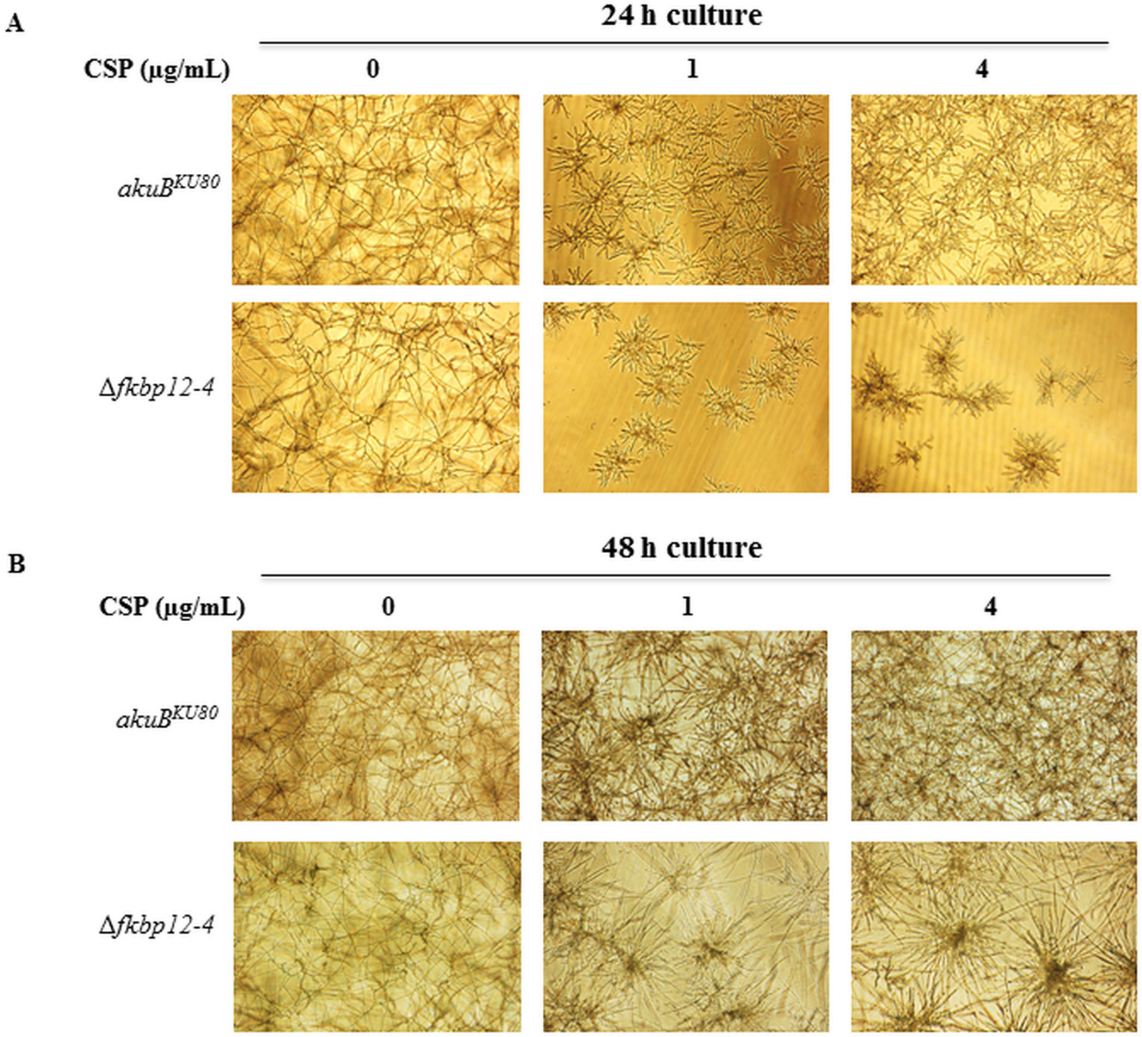
Δ*fkbp12-4* is more susceptible to caspofungin and lacks paradoxical growth at higher concentrations of caspofungin. **(A)** *A*. *fumigatus akuB* ^*KU80*^ and the Δ*fkbp12-4* conidia (10^4^/mL) were cultured in RPMI for 24 hours either in the absence or presence of 1 μg/ml and 4 μg/ml caspofungin (CSP). **(B)** *A*. *fumigatus akuB* ^*KU80*^ and the Δ*fkbp12-4* conidia (10^4^/mL) cultured in RPMI for 48 hours either in the absence and presence of 1 μg/ml and 4 μg/ml caspofungin (CSP).

Because the Δ*fkbp12-4* strain showed reduced growth in comparison to the wild-type strain, we also examined the effect of caspofungin, an anti-cell wall antifungal agent, on all the FKBP12 deletion strains. At 1 μg/mL caspofungin, Δ*fkbp12-1*, Δ*fkbp12-2*, Δ*fkbp12-3*, and Δ*fkbp12-1*Δ*fkbp12-2* strains demonstrated similar susceptibility to caspofungin, while Δ*fkbp12-4* demonstrated increased susceptibility (Fig 4D). As is normally observed in the wild-type strain, paradoxical growth effect was noted at higher caspofungin concentrations in all deletion strains except for the Δ*fkbp12-4* strain (Fig 4E) [62–64]. In the presence of the combination of FK506 and caspofungin, the Δ*fkbp12-1* and Δ*fkbp12-1*Δ*fkbp12-2* strains demonstrated slightly increased growth compared to other deletion strains as well as the wild-type strain. The growth of Δ*fkbp12-1* and Δ*fkbp12-1*Δ*fkbp12-2* strains in the presence of both drugs (FK506+caspofungin) was indistinguishable from their growth in response to caspofungin alone (Fig 4F). To more clearly visualize the inhibition of paradoxical growth at higher caspofungin concentrations in the Δ*fkbp12-4* strain, the Δ*fkbp12-4* strain was cultured in RPMI liquid media supplemented with caspofungin at 1 μg/mL and 4 μg/mL (Fig 5A and 5B). In contrast to the *akuB* ^*KU80*^ strain the Δ*fkbp12-4* did not demonstrate paradoxical growth recovery. This lack of paradoxical growth in Δ*fkbp12-4* may be due to the fact that the Δ*fkbp12-4* showed reduced growth rate in comparison to the wild-type strain and the other FKBP12 deletion strains.

**Fig 6:**
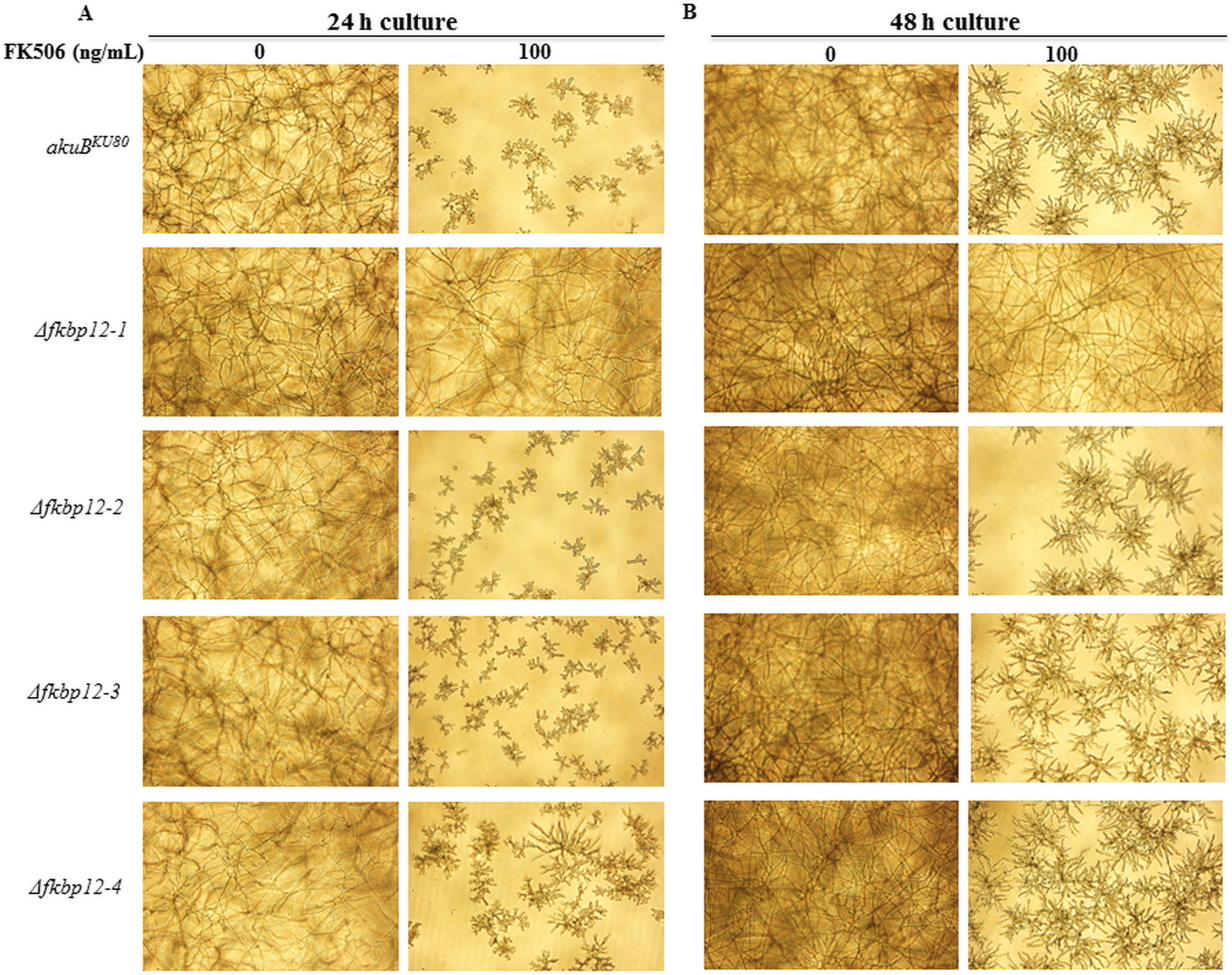
Δ*fkbp12-1* demonstrates full hyphal growth in response to FK506; Δ*fkbp12-4* is slightly tolerant to FK506. **(A)** *A*. *fumigatus* conidia (10^4^/mL) incubated in RPMI for 24 hours. **(B)** *A*. *fumigatus* conidia (10^4^/mL) incubated in RPMI with 100 ng/mL FK506 for 24 hours. **(C)** *A*. *fumigatus* conidia (10^4^/mL) incubated in RPMI for 48 hours. **(D)** *A*. *fumigatus* conidia (10^4^/mL) incubated in RPMI with 100 ng/mL FK506 for 48 hours.

To visualize hyphal growth in response to FK506, the single deletion strains were also cultured in liquid media supplemented with FK506 (Fig 6). The Δ*fkbp12-1* displayed full hyphal growth in the presence of FK506 after 24 hours, while *Δfkbp12-4* seemed slightly tolerant in comparison to the other deletion strains (Fig 6A). At 48 hours the respective strains demonstrated improved growth, although the inhibitory effect of FK506 was still evident in all except for the Δ*fkbp12-1* strain (Fig 6B). The Δ*fkbp12-1*Δ*fkbp12-2* strain was also resistant to FK506 (data not shown).

### FKBP12-1 localizes to the cytoplasm and nuclei but also shifts to the hyphal septa following exposure to FK506

**Fig 7:**
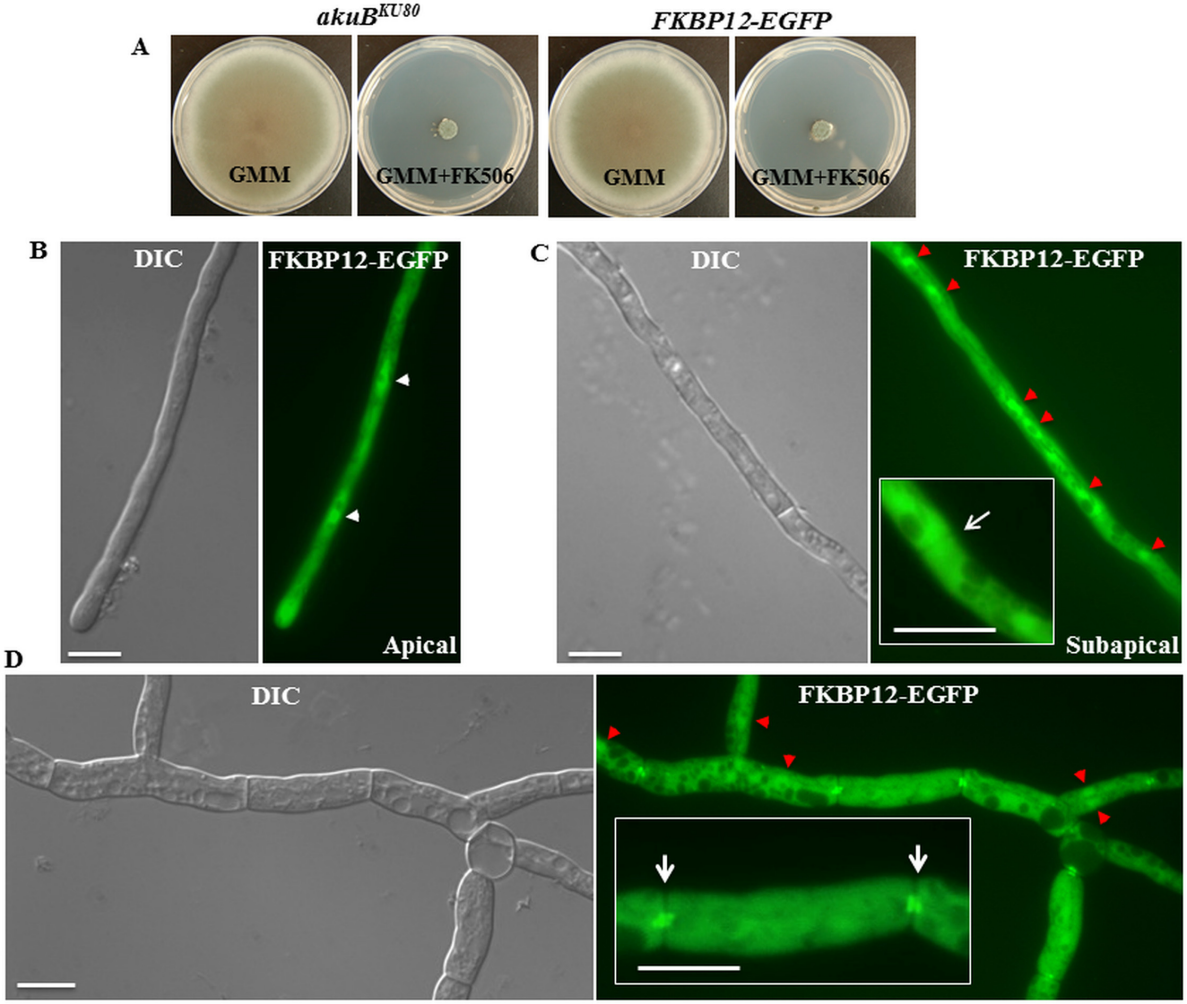
FK506 altered the localization of FKBP12-1 to the hyphal septum. **(A)** Functionality of the expressed FKBP12-1-EGFP was assessed by comparing the growth of the FKBP12-1-EGFP expression strain with the *akuB* ^*KU80*^ strain either in the absence or presence of FK506 (0.1 μg/mL) **(B, C)** Under normal growth conditions, FKBP12-1 evenly distributes throughout the cytoplasm and is also found in the nucleus at the hyphal tips (panel B) and sub-apical compartment (panel C and panel D) (marked by a white arrow heads in panel B and by red arrowheads in panel C and panel D). It is not seen at the septum (marked by a white arrow in the Fig 7C inset). **(C)** In the presence of FK506, FKBP12-1 can be seen localized as a double bar on either side of the septa indicating its binding to calcineurin complex at the hyphal septum (marked by a white arrows in the Fig 7D inset).

**Fig 8:**
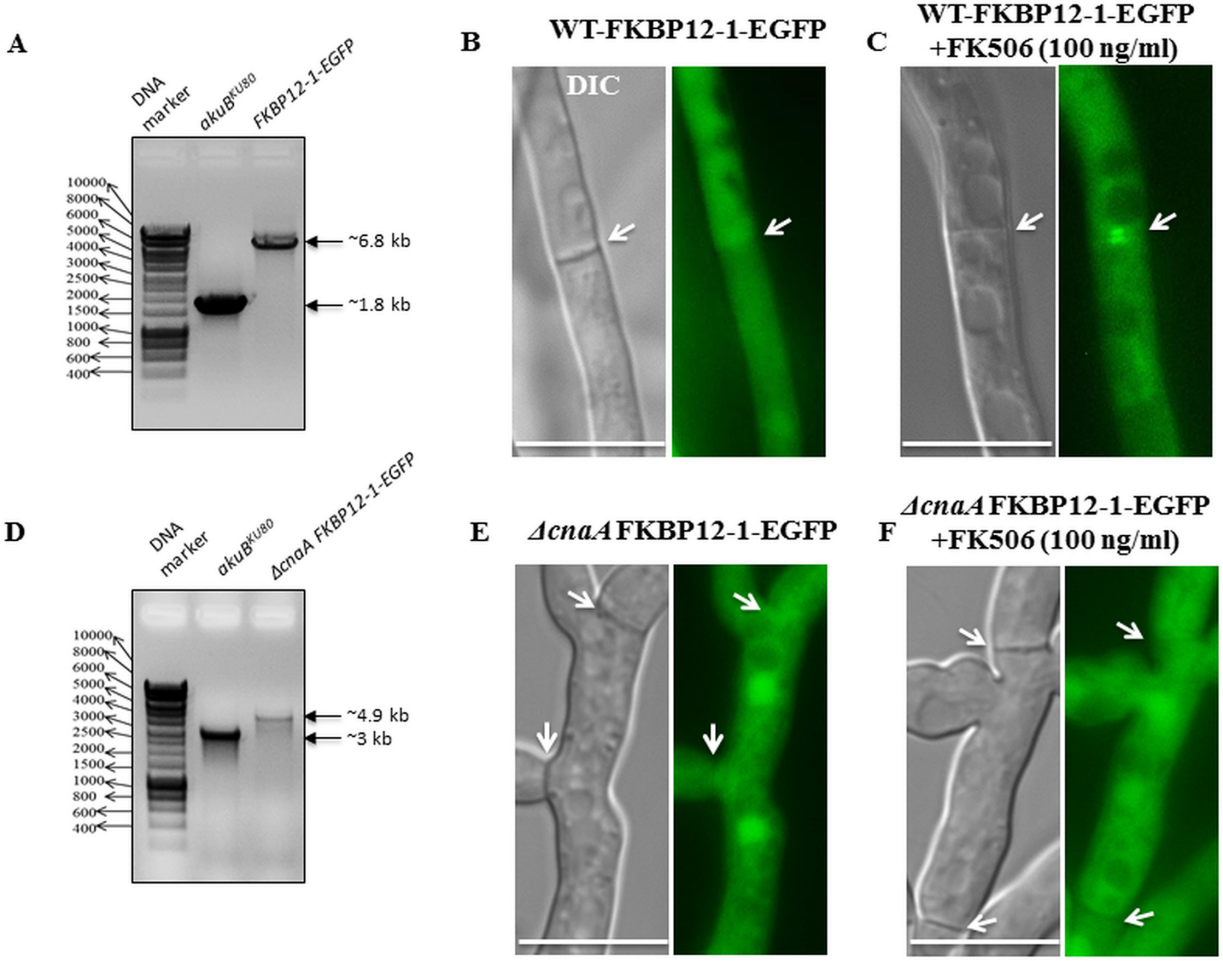
FKBP12-1 localizes to the hyphal septum through binding to CnaA in the presence of FK506. **(A)** Confirmation of generation of the FKBP12-1-EGFP expression strain by PCR and fluorescence microscopy. **(B)** Cytosolic localization of FKBP12-1-EGFP. **(C)** Septal localization of FKBP12-1-EGFP in the presence of FK506 (indicated by white arrows). **(D)** Confirmation of generation of the *cnaA* deletion in the FKBP12-1-EGFP expression background strain by PCR and fluorescence microscopy. **(E)** Cytosolic localization of FKBP12-1-EGFP in the calcineurin null strain. **(F)** Absence of septal localization of FKBP12-1-EGFP in the calcineurin null strain in the presence of FK506 (indicated by white arrows).

Previous studies from our laboratory revealed the localization of calcineurin complex at the hyphal septum in a disc-like manner around the septal pore [32, 33]. We took advantage of this localization to verify the binding of FKBP12-1 to calcineurin in the presence of FK506. In order to examine the localization of FKBP12-1 and confirm its association with calcineurin in the presence of FK506, a strain expressing FKBP12-1 tagged to EGFP (*fkbp12-1-egfp*) at its native locus was generated. Homologous recombination was confirmed by PCR (data not shown). To confirm functionality of the tagged FKBP12-1 protein, radial growth assays and testing with FK506 were performed. No difference between the wild type and the FKBP12-1-EGFP strains were noted (Fig 7A). Under normal growth conditions, FKBP12-1 was localized evenly throughout the cytoplasm and also in the nuclei at the hyphal tips and in the sub-apical compartments (Fig 7B and 7C). Upon exposure to FK506, FKBP12-1 also localized to the septa in the form of a disc-like pattern as noted earlier with calcineurin (Fig 7D; see inset image), suggesting its binding to calcineurin and inhibition of calcineurin activity at the hyphal septum as previously reported [32]. To confirm this, we next constructed an FKBP12-1-EGFP expression strain and deleted the catalytic subunit of calcineurin encoding gene *cnaA* in this background (Fig 8A–8C and 8D). Akin to what was seen earlier with the wild type FKBP12-1-EGFP, the localization patterns of FKBP12-1 in a calcineurin null strain (*fkbp12-1-egfp*Δ*cnaA*) revealed nuclear and cytoplasmic localization under basal conditions (Fig 8E). However, upon exposure to FK506 FKBP12-1-EGFP failed to localize to the septa, confirming that FKBP12-1 localizes at the hyphal septum through binding to calcineurin upon exposure to FK506 (Fig 8F).

**Fig 9:**
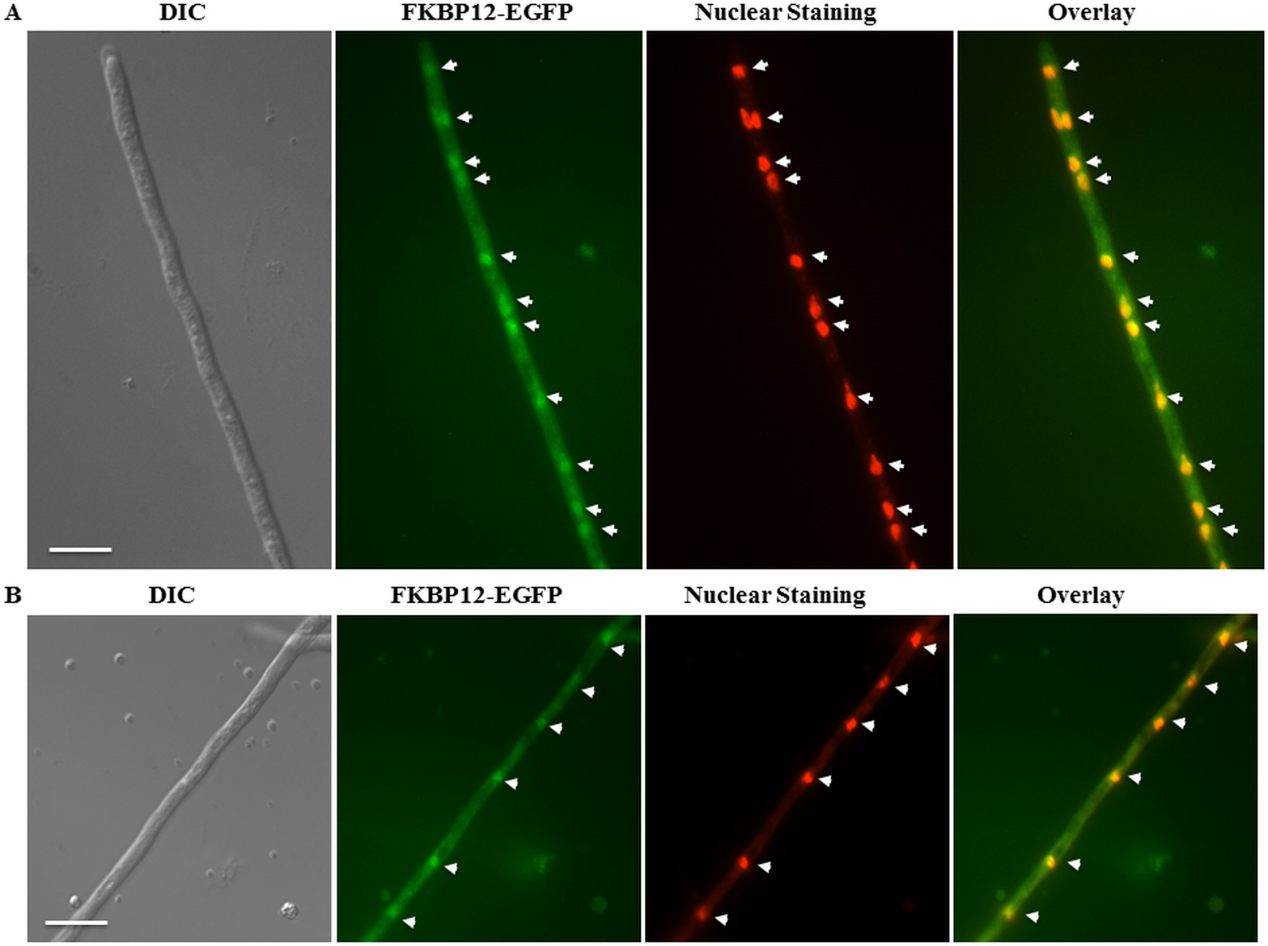
FKBP12-1 is seen in the cytoplasm and in the nuclei under basal conditions. **(A, B)** Propidium iodide staining confirms FKBP12-1 localization to the nucleus in the apical compartment (Fig 9A; marked by white arrow heads) and the sub-apical compartment (Fig 9B; marked by white arrow heads).

Nuclear localization of FKBP12-1 was confirmed by propidium iodide staining of nuclei (Fig 9). Although we could not identify any nuclear localization signal consensus sequence in FKBP12-1, we speculate that FKBP12-1 might translocate into the nucleus by binding to other protein/s.

### FKBP12-1 proteins do not play a key role in virulence

**Fig 10:**
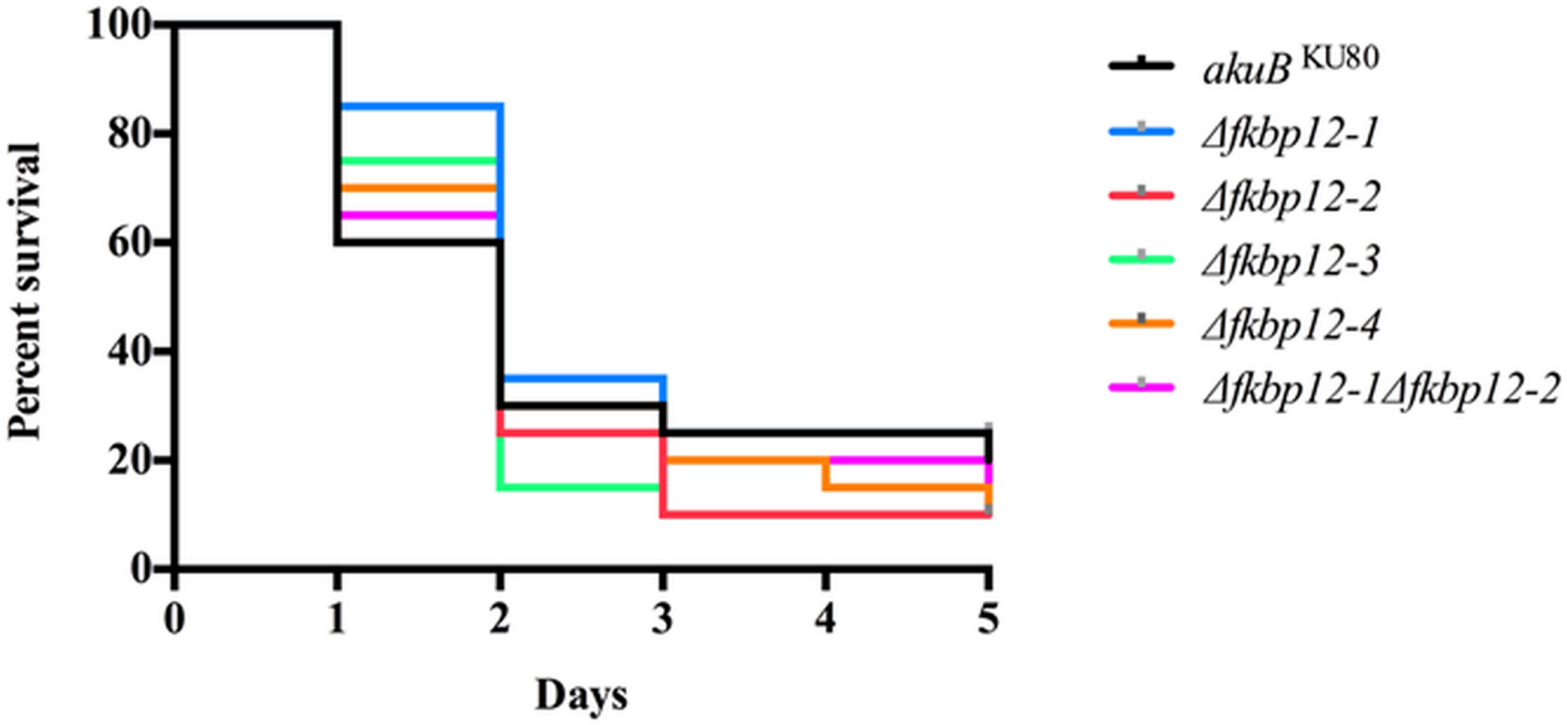
Deletion of FKBP12 encoding genes did not alter virulence of *A*. *fumigatus*. Larvae of the wax moth *Galleria mellonella* were injected with 5 μl of 1 x 10^8^ spores/ml (a total inoculum of 2 x 10^5^ spores) of the wild type, Δ*fkbp12-1*, Δ*fkbp12-2*, Δ*fkbp12-3*, Δ*fkbp12-4*, and*Δfkbp12-1Δfkbp12-2* strains. 20 larvae were included in each arm. Infected larvae were incubated at 37°C with survival scored daily for 5 days.

Earlier reports on the human pathogenic bacterium, *Legionella pneumophila*, and the human parasitic protozoan, *Trypanosoma cruzi*, have revealed the association of the FKBP12 proteins with virulence [65, 66]. While in the plant pathogenic fungus *Botrytis cinerea* disruption of the only ortholog of *FKBP12*, *BcPIC5*, caused a reduction in pathogenicity [50], in another plant pathogen *Fusarium graminearum* the interaction of FKBP12 with a virulence factor FGL1 encoding a secreted lipase was demonstrated [67]. In order to verify if *A*. *fumigatus* FKBP12s played a role in virulence, we employed a screening systemic aspergillosis infection model using the heterologous invertebrate host *Galleria mellonella*. Infection of the larvae with all the FKBP12 deletion strains led to survival comparable to that seen in the wild-type strain (p = 0.64) (Fig 10). No difference in melanization of the *Galleria*, which serves as an indication of immune response, was noted following infection with the wild type strain or FKBP12 deletion strains.

## Discussion

Previous work has demonstrated the potential of drugs currently used as immunosuppressants, such as FK506, as possible therapeutic agents against invasive fungal infections, including *A*. *fumigatus* [11–13, 68, 69]. While in humans the FK506-FKBP12 complex binding to calcineurin suppresses the immune system, in *A*. *fumigatus* binding of the FK506-FKBP12 complex to fungal calcineurin leads to impaired growth and virulence [9, 28]. Thus, drugs like FK506 could be chemically modified or repurposed for targeted inhibition of fungal-specific calcineurin in the treatment of invasive fungal infections. While study of FKBP12, one of the key proteins through which FK506 and rapamycin exert their effects, has been extensive in humans, work in fungi has been limited [50, 51, 67]. Deletion of orthologs of FKBP12 have been found to mediate resistance to FK506 in pathogenic fungi, including *C*. *neoformans* and *M*. *circinelloides* [52, 53, 55, 70]. However, studies in *A*. *fumigatus*, a leading cause of death secondary to invasive fungal infection as well as the pathogen with the largest financial burden of all invasive fungal infections [70], have not yet been undertaken [61]. In this study, we characterized the four putative *A*. *fumigatus* FKBP12 orthologs through deletion analysis coupled with phenotypic and virulence studies, and identified FKBP12-1 as responsible for binding to FK506 and inhibiting calcineurin and FKBP12-4 as involved in basal growth.

Given that FKBP12-1 is the ortholog with the most sequence similarity to human FKBP12, it is not surprising that deletion of FKBP12-1 encoding gene led to FK506 resistance, presumably through a lack of binding to FK506. Septal localization pattern of FKBP12-1 only in the presence of FK506 in the wild-type but not in the *cnaA* deletion background further supports the hypothesis that loss of binding to FK506 is responsible for the resistance. Localization under basal conditions to the cytoplasm and nucleus is consistent with FKBP localization in other organisms [61], while the presence of the protein at the septa in the presence of FK506 in the FKBP12-1-EGFP strain but not in the FKBP12-1-EGFPΔ*cnaA* strain suggests calcineurin-binding and inhibition [33]. However, three of the 14 residues important for binding to FK506 are different from human FKBP12 in the *A*. *fumigatus* FKBP12-1 protein. On the other hand, FKBP12-2 and FKBP12-3 are mutated at three and four of the 14 residues, respectively, and deletion of these proteins does not lead to resistance to FK506. Thus, the impact of residue changes in FKBP12-2 and FKBP12-3 on binding to FK506 is unclear–it is possible that binding to FK506 is retained despite these changes. Alternatively, the FKBP12-2 and FKBP12-3 proteins may not be endogenously expressed in the fungus and the susceptibility to FK506 observed may be the result of the actions of FKBP12-1 alone on calcineurin, a hypothesis supported by the resistance to FK506 seen in the Δ*fkbp12-1*Δ*fkbp12-2* deletion strain. Interestingly, FKBP12-4, which shares the least sequence similarity to human FKBP12 but also differs at only two of the 14 residues noted to be involved in FK506 binding, shows minimal tolerance to FK506. This suggests that perhaps its mutations lessen but do not preclude binding to FK506. Alternatively, it is possible that binding to FK506 remains intact but the long N-terminal region of the protein interferes with binding of the FKBP12-FK506 complex to calcineurin. However, the tolerance is difficult to interpret as far as biologic relevance in the face of the observed growth defect in Δ*fkbp12-4* strain.

Taken together, these results suggest that homology to human FKBP12 can be predictive in determining resistance to FK506, but they also suggest that the number of residues mutated at the 14 residues previously found to be critical for binding is less predictive. Indeed, while FKBP12-4 has the fewest number of mutations in residues involved in FK506 binding, only the Δ*fkbp12-1* strain, that which still retains the the most sequence similarity to human FKBP12, is resistant to FK506. Most significantly, the present data suggest that even with differences from human FKBP12 that include alterations in polarity and size, binding of FKBP12-1 to FK506 can still occur. Therefore, designing a FK506 analog that will fit into the altered binding pocket of the fungal FKBP12-1, but not into the binding pocket of the human FKBP12, should be explored.

In addition to determining the *A*. *fumigatus* FKBP12 responsible for mediating resistance to FK506, we sought to better understand the role of this important family of proteins in fungal biology and thus characterized all four deletion strains as well as a double deletion strain (Δ*fkbp12-1*Δ*fkbp12-2)*. As FKBP12s have previously been found to be dispensable for growth in other fungi and model organisms, except under some stress conditions, it is not surprising that the Δ*fkbp12-1*, Δ*fkbp12-2*, Δ*fkbp12-3*, and Δ*fkbp12-1*Δ*fkbp12-*2 strains all demonstrated radial growth consistent with that seen in the wild-type strain [19, 20, 45, 46]. While the Δ*fkbp12-1* strain did have a statistically significant difference in growth compared to wild type (p = 0.0405), that the Δ*fkbp12-1*Δ*fkbp12-2* strain did not, and that the Δ*fkbp12-1* strain did reach full growth by the end of the 5 day incubation period, suggest a lack of biologic relevance.

Unexpectedly, we found that the FKBP with the least sequence similarity to human FKBP12, FKBP12-4, plays a role in growth of the pathogen under basal conditions. The Δ*fkbp12-4* strain displayed universally slow growth throughout the 5 day testing period. The FKBP12-4 protein is significantly larger than the other three FKBP12s (489 amino acids versus 112 to 134 amino acids in FKBP12-1 through FKBP12-3), with an extended N-terminal sequence.

Though more comparable in size to the other *A*. *fumigatus* FKBP12s, human FKBP12.6, an isoform of human FKBP12, has an N-terminal sequence important for binding to the ryanodine receptor [71]. Perhaps the long N-terminal sequence of *A*. *fumigatus* FKBP12-4 may similarly have residues important for binding to a target, which in this case is related to growth regulation or cell wall stability. It is also possible that the growth defect is a result of changes in amino acids in the regions FKBP12-4 shares with the other *A*. *fumigatus* FKBPs. As FKBPs are involved in protein folding, FKBP12-4 may be required for efficient folding of a protein important to growth in *A*. *fumigatus*. Regardless, the probable reason for this growth defect may be related to a defect in cell wall integrity.

The role of FKBPs in the pathogenesis was also studied through the use of *G*. *mellonella*, which has been shown to be a reliable screening model of infection in *A*. *fumigatus* [72, 73]. There was no statistically significant difference in virulence between the FKBP12 deletion and wild-type strains.

In conclusion, we have identified four *A*. *fumigatus* FKBP12 orthologs, and through deletion analysis confirmed FKBP12-1 binding to FK506 in *A*. *fumigatus*. We have also established that FKBP12-4 leads to a growth defect under basal conditions. Future directions for this work include further study of FKBP12-1, specifically via more in depth identification of fungal-specific residues most important for binding to FK506 through targeted mutagenesis.

## Supporting Information

S1 Fig Multiple sequence alignment comparing *A*. *fumigatus* Fkbp12 proteins to those in other organisms. Multiple sequence alignment was performed comparing orthologous FKBP12 proteins from Human (HsFKBP12), *S*. *cerevisiae* (ScFkbp12), *C*. *neoformans* (CnFkbp12), and *A*. *fumigatus* (FKBP12-1, FKBP12-2, FKBP12-3 and FKBP12-4) using ClustalW (http://www.ebi.ac.uk/Tools/msa/clustalw2/).
(TIF)

S1 Table Primers Used in the Generation of Deletion Strains. (DOCX)

S2 Table Primers Used for PCR Verification of Deletion Strains. (DOCX)

S3 Table Primers Used for Generation of Probes for Southern. (DOCX)

S4 Table Primers Used in the Generation of the EGFP Strains. (DOCX)

S5 Table Primers Used for Verification of Fkbp12-1-EGFP and Fkbp12-1ΔCnaA Strains. (DOCX)
