# Supplementary figures and images for: Characterization of the FKBP12-Encoding Genes in Aspergillus fumigatus
Source: PLoS One. 2015 Sep 14;10(9):e0137869. doi: 10.1371/journal.pone.0137869 (PMC4569257; doi:10.1371/journal.pone.0137869)

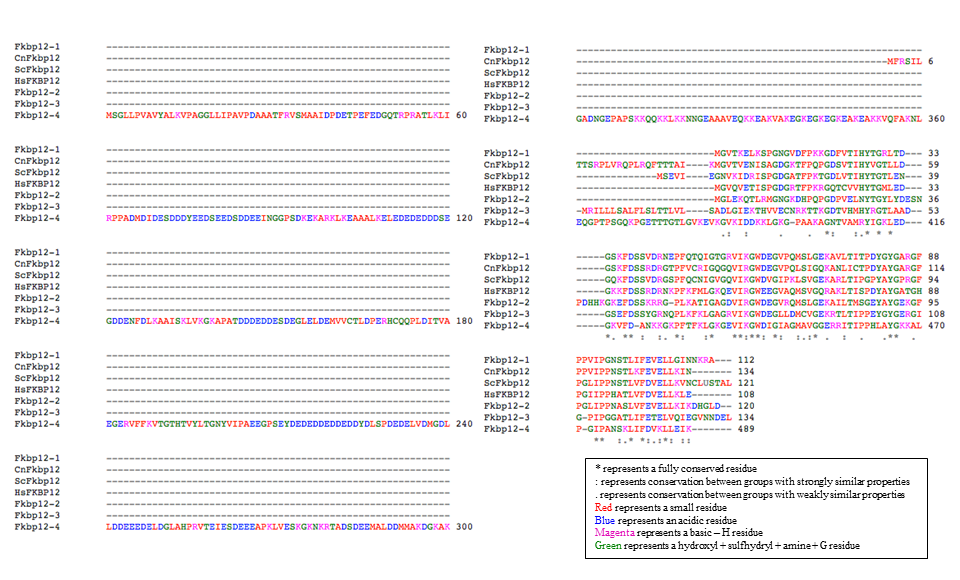

Supplement: S1 Fig — Multiple sequence alignment was performed comparing orthologous FKBP12 proteins from Human (HsFKBP12), S. cerevisiae (ScFkbp12), C. neoformans (CnFkbp12), and A. fumigatus (FKBP12-1, FKBP12-2, FKBP12-3 and FKBP12-4) using ClustalW (http://www.ebi.ac.uk/Tools/msa/clustalw2/). (TIF) [file pone.0137869.s001.tif]
