# Supplementary material for: Characterization of the FKBP12-Encoding Genes in Aspergillus fumigatus
Source: PLoS One. 2015 Sep 14;10(9):e0137869. doi: 10.1371/journal.pone.0137869 (PMC4569257; doi:10.1371/journal.pone.0137869)
Supplement: S1 Table — (DOCX) [file pone.0137869.s002.docx]

**S1 Table: Primers Used in the Generation of Deletion Strains**

| Name | Sequence (5’-3’) | Direction |
| --- | --- | --- |
| ***Δfkbp12-1*** | ATGCGGATCCcgctatttaccggtgggagcgaag  GCTAGAATTCtgtgattttctggtggagaataga  ATGCGAATTCGCTGATCCTATGGATCTCAGAACA  GCTAGAGCTCcgaacctgtcatatcaacccctgg  cgctatttaccggtgggagcgaagcggtcaa  cgaacctgtcatatcaacccctggcggta | Forward  Reverse  Forward  Reverse  Forward  Reverse |
| Fkbp12-1-promo-BamHI-F  Fkbp12-1-promo-EcoRI -R  pyrG-EcoRI-F  Fkbp12-1-term-SacI-R  Fkbp12-1-promo-F  Fkbp12-1-term-R |  |  |
| ***Δfkbp12-2*** | ATGCGTCGACGAGAAATATGCAGACCAGATCC  GCTAGAATTCTATATCCTATGCCTATTCCTGT  ATGCGGATCCggttgatctgggaggtgatcact  GTCAgcggccgcCaacgactagtactgacgatgac | Forward  Reverse  Forward  Reverse |
| Fkbp12-2-promo-SalI-F  Fkbp12-2-promo-EcoRI–R  Fkbp12-2-term-BamHI-F  Fkbp12-2-term-NotI-R |  |  |
| ***Δfkbp12-3*** | ATGCGCGGCCGCGCCACTTGGATGATTTGGGACA  GCGCTCTAGATTTGAATCTTATAGATGATGCGAA  GCGCGAATTCGTCTTTGGATGGAAGGCCATATAA  GCTAGTCGACTACAGCGAGGAAGGCGATTTTGGA | Forward  Reverse  Forward  Reverse |
| Fkbp12-3-promo-NotI-F  Fkbp12-3-promo-Xbal–R  Fkbp12-3-term-EcoRI-F  Fkbp12-3-term-SalI-R |  |  |
| ***Δfkbp12-4*** | ATGCGTCGACaGGTGAGAGAGCTGGCAATGATAT  GCGCGAATTCTTTGATTGAATGCAGGTAGCGAAA  ATGCgcggccgcGCAGCTCTGTAGCCTGCTTGT  ATGCGAGCTCGATACAGCAGGATTCGTTTGAGGG | Forward  Reverse  Forward  Reverse |
| Fkbp12-4-promo-SalI-F  Fkbp12-4-promo-EcoRI-R  Fkbp12-4-term-NotI-F  Fkbp12-4-term-SacI-R |  |  |
| ***Δfkbp12-1Δfkbp12-2*** | ATGCAAGCTTGAGATTGACTGGATTCGGC  ATGCCCTGCAGGTATATCCTATGCCTATTC  ATGCGATATCGGTTGATCTGGGAGGTGATC  GATAGCGGCCGCTGACGATGACAGCCTCAA | Forward  Reverse  Forward  Reverse |
| Fkbp12-2-promo-HindIII-F  Fkbp12-2-promo-SbfI-R  Fkbp12-2-term-EcoRV-F  Fkbp12-2-term-NotI-R |  |  |
