# Supplementary material for: Characterization of the FKBP12-Encoding Genes in Aspergillus fumigatus
Source: PLoS One. 2015 Sep 14;10(9):e0137869. doi: 10.1371/journal.pone.0137869 (PMC4569257; doi:10.1371/journal.pone.0137869)
Supplement: S2 Table — (DOCX) [file pone.0137869.s003.docx]

**S2 Table: Primers Used for PCR Verification of Deletion Strains**

| Name | Sequence (5’-3’) | Direction |
| --- | --- | --- |
| ***Δfkbp12-1*** | CGAAGACCTACTTTCGAATCTCG  GATTGTTGGATGCTGAAGATGG | Forward  Reverse |
| Fkbp12-1-promo-flank-F  Fkbp12-1-term-flank-R |  |  |
| ***Δfkbp12-2*** | CGGACATGCCAAAGACTCAC  GGCAGACTGGCAAAAGTACC  ATGGGCCTCGAAAAACAAAC  CTAATCAAGACCATGGTCC | Forward  Reverse  Forward  Reverse |
| Fkbp12-2-promo-flank-F  Fkbp12-2-term-flank-R  Fkbp12-2-F–WT  Fkbp12-2-R-WT |  |  |
| ***Δfkbp12-3*** | GGATGATTTGGGACACGA  CGAGGAAGGCGATTTTGG  ATGCGCATTCTACTTCTCTCC  CTACAGCTCATCATTATTAACCC | Forward  Reverse  Forward  Reverse |
| Fkbp12-3-promo-F*  Fkbp12-3-term-R*  Fkbp12-3-F-WT  Fkbp12-3-R-WT  *also used for linearization |  |  |
| ***Δfkbp12-4*** | ATTCGCCTGGCTTAGAAGTC  CGACGAGTATCAAAGGTGGA  ATGTCCGGTCTTCTGCCTGT  TTATTTGATCTCAAGGAGTTTAACG | Forward  Reverse  Forward  Reverse |
| Fkbp12-4-promo-flank-F  Fkbp12-4-term-flank-R  Fkbp12-4-WT-F  Fkbp12-4-W-R |  |  |
| ***Δfkbp12-1Δfkbp12-2*** | CGGACATGCCAAAGACTCAC  GGCAGACTGGCAAAAGTACC  ATGGGCCTCGAAAAACAAAC  CTAATCAAGACCATGGTCC | Forward  Reverse  Forward  Reverse |
| Fkbp12-2-promo-flank-F  Fkbp12-2-term-flank-R  Fkbp12-2-F-WT  Fkbp12-2-R-WT |  |  |
