# Supplementary material for: Characterization of the FKBP12-Encoding Genes in Aspergillus fumigatus
Source: PLoS One. 2015 Sep 14;10(9):e0137869. doi: 10.1371/journal.pone.0137869 (PMC4569257; doi:10.1371/journal.pone.0137869)
Supplement: S3 Table — (DOCX) [file pone.0137869.s004.docx]

**S3 Table: Primers Used for Generation of Probes for Southern**

| Name | Sequence (5’-3’) | Direction |
| --- | --- | --- |
| ***Δfkbp12-1*** | CGAGAAGAAGGCACACGG  CTTGGCGCCTTGATTCAA | Forward  Reverse |
| Fkbp12-1-probe-F  Fkbp12-1-probe-R |  |  |
| ***Δfkbp12-2*** | CGAATCTCCTCTCTCGCC  GCCTGTACCTATCAGCAT | Forward  Reverse |
| Fkbp12-2-probe-F-upstream  Fkbp12-2-probe-R-downstream |  |  |
| ***Δfkbp12-3*** | GAACCGTCCTTGCTGCATT  GCGAATGAAGGAATTGGA | Forward  Reverse |
| Fkbp12-3-probe-F  Fkbp12-3-probe-R |  |  |
| ***Δfkbp12-4*** | CGGTCTTGCCGTCCAAAT  CTCCAAGCTTTTGGGACTG | Forward  Reverse |
| Fkbp12-4-probe-F  Fkbp12-4-probe-R |  |  |
| ***Δfkbp12-1Δfkbp12-2*** | GACAAAGTTCGACGCCTTC  GAAGAAGCCACGCGCCTT | Forward  Reverse |
| Fkbp12-2-probe-F-double  Fkbp12-2-probe-R-double |  |  |
