# Supplementary material for: Characterization of the FKBP12-Encoding Genes in Aspergillus fumigatus
Source: PLoS One. 2015 Sep 14;10(9):e0137869. doi: 10.1371/journal.pone.0137869 (PMC4569257; doi:10.1371/journal.pone.0137869)
Supplement: S4 Table — (DOCX) [file pone.0137869.s005.docx]

**Supplemental Table 4: Primers Used in the Generation of the EGFP Strains**

| Name | Sequence (5’-3’) | Direction |
| --- | --- | --- |
| ***fkbp12-1-egfp*** | CAAGGGTACCTTATCGTGATCC  ATGCGGATCCAGCCCTCTTGTTGTTGATG  ATGCCCTGCAGGATACCTGCAAATGATATG  ATGCAAGCTTCCACGTCGAACCTGTC  ATGGGTGTCACCAAGGAACT | Forward  Reverse  Forward  Reverse  Forward |
| Fkbp12-1-gene-F-KpnI-gfp  Fkbp12-1-gene-R-Bam-H1-gfp  Fkbp12-1-T-F-SbfI-gfp  Fkbp12-1-T-R-HindIII-gfp  Fkbp12-F |  |  |
| ***ΔCnaA*** | GTACGGTACCAAGTAGTCACCTGCGTGGACGTGG  GTGGAAAGCTTtggttggatagacaacggcac  CGACGGATCCATGGATCAAGCACTGGCG  CGACGGATCCGGCTTCCCTAGTCTC | Forward  Reverse  Forward  Reverse |
| CnaA-promo-KpnI-F  CnaA-term-HindIII-R  GCNA-F2  GCNA-R-Bam |  |  |
