# Supplementary material for: Characterization of the FKBP12-Encoding Genes in Aspergillus fumigatus
Source: PLoS One. 2015 Sep 14;10(9):e0137869. doi: 10.1371/journal.pone.0137869 (PMC4569257; doi:10.1371/journal.pone.0137869)
Supplement: S5 Table — (DOCX) [file pone.0137869.s006.docx]

**Supplemental Table 5: Primers Used for Verification of Fkbp12-1-EGFP and Fkbp12-1ΔCnaA Strains**

| Name | Sequence (5’-3’) | Direction |
| --- | --- | --- |
| **For Sequencing** | CAAGGGTACCTTATCGTGATCC  ATGCGGATCCAGCCCTCTTGTTGTTGATG  ATGCCCTGCAGGATACCTGCAAATGATATG  ATGCAAGCTTCCACGTCGAACCTGTC | Forward  Reverse  Forward  Reverse |
| Fkbp12-1-gene-F-KpnI-gfp  Fkbp12-1-gene-R-Bam-H1-gfp  Fkbp12-1-T-F-SbfI-gfp  Fkbp12-1-T-R-HindIII-gfp |  |  |
| **For PCR Confirmation** | CCAATGACAAGCTGCAATGGTG  TGTTGGATGCTGAAGATGGAAG  AAGTCCTCGTGTACTGTGTAAGCG  CACAAGTTCAGCGTGT | Forward  Reverse  Forward  Reverse |
| Fkbp12-1-GFP-flank-F  Fkbp12-1-GFP-flank-R  Hyg-F-Screen  pUCGH-R |  |  |
